# Duodenal Gangliocytic Paraganglioma Requiring a Pancreaticoduodenectomy: A Case Report and Review of the Literature

**DOI:** 10.1155/2018/6292789

**Published:** 2018-09-26

**Authors:** Laurie Adams, Theodore M. Friedman, Timothy R. Shaver, George Younan

**Affiliations:** ^1^Department of Surgery, Inova Fairfax Hospital, Fairfax, VA, USA; ^2^Department of Pathology, Inova Fair Oaks Hospital, Fairfax, VA, USA; ^3^Division of Hepato-Pancreato-Biliary Surgery, Virginia Surgery Associates, Fairfax, VA, USA

## Abstract

**Introduction:**

Duodenal gangliocytic paragangliomas (GPs) are a subclass of duodenal neuroendocrine neoplasms and are exceedingly rare. They have been associated with an indolent behavior; however, they can rarely metastasize. Radical resection like a pancreaticoduodenectomy is sometimes indicated. We hereby present a case requiring major surgery and perform a literature search about this disease.

**Presentation of Case:**

A 49-year-old Caucasian female, who presented with an upper gastrointestinal bleed, was found to have a hypodense mass in the second/third portion of the duodenum. A biopsy of the mass during upper endoscopy was inconclusive. A pancreaticoduodenectomy was recommended based on the high suspicion for a duodenal adenocarcinoma and was performed successfully. Her final pathology revealed a duodenal gangliocytic paraganglioma.

**Discussion:**

The majority of duodenal GPs present as gastrointestinal bleeds while others less commonly present with anemia, abdominal pain, duodenal obstructive symptoms, pancreatitis, or abnormal incidental findings on axial abdominal imaging. Duodenal GPs were initially viewed as benign tumors of the duodenum; however, there have been increasing incidence reports of hematogenous and lymphatic metastasis. Appropriate treatment of duodenal GPs is still controversial and ranges from local endoscopic submucosal resection to major radical surgery.

**Conclusion:**

Duodenal GPs are very rare tumors of the second portion of the duodenum presenting with upper gastrointestinal bleeding and local symptoms of surrounding organs. Local or radical resection is usually recommended to prevent bleeding and the minor risk of metastatic spread.

## 1. Introduction

Duodenal neuroendocrine tumors (NETs) are infrequent, comprising about 1–3% of gastrointestinal endocrine tumors [1, 2]. Duodenal gangliocytic paragangliomas are a subclass of duodenal neuroendocrine neoplasms, ranking third in frequency behind gastrinomas and somatostatinomas of the duodenum, comprising about 10% of those, and most commonly arising from the second portion of the duodenum [3]. GPs were first described more than seventy years ago, and since then, they have been reported as small series or case reports in about two hundred publications [4]. Although thought to be benign entities, reports have demonstrated their malignant potential, either by spread to regional lymph nodes or metastasis to distant organs [5–7]. The World Health Organization (WHO) published a new classification of gastrointestinal neuroendocrine tumors in 2010 based on their mitotic activity and their Ki-67 proliferation index, regardless of their site of origin, so as to classify them into low-grade, intermediate-grade, and high-grade tumors [8–10]. Duodenal GPs should be differentiated from low-grade NET as they have a more indolent clinical behavior and improved prognosis [11–13]. We hereby present a case of upper gastrointestinal bleeding from an ulcerated duodenal mass, requiring a pancreaticoduodenectomy, and found to be a duodenal GP on final pathology examination.

## 2. Case Presentation

**Figure 1:**
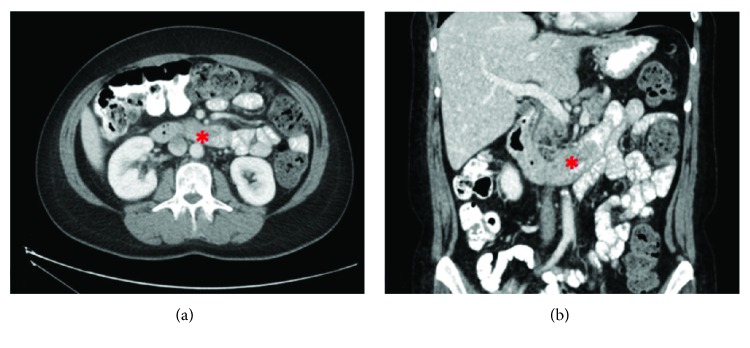
Axial (a) and coronal (b) computed tomography images of the duodenal GP, appearing as a hypodense lesion, shown to arise from the second/third portion of the duodenum and abut the uncinate process of the pancreas. Tumor is marked by an asterisk.

**Figure 2:**
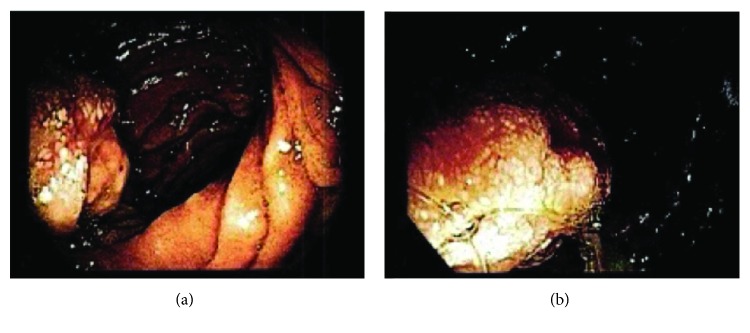
Endoscopic images of the large ulcerated mass found in the second/third portion of the duodenum. There was no bleeding stigmata at the time of endoscopy.

A 49-year-old Caucasian woman presented to the emergency room with three-day history of palpitations, shortness of breath, pallor, and black tarry stools. She was found to be anemic with hemoglobin of 4.4 g/dL. She was transfused, and a gastrointestinal bleeding workup was initiated. The rest of the physical examination and additional blood tests were within normal limits. A computed tomography (CT) scan of the abdomen showed a possible hypodense mass in the second/third portion of the duodenum that is intraluminal with an extraluminal component abutting the uncinate process of the pancreas. There was no sign of any other disease in the abdomen and lower chest (Figure 1). Colonoscopy was negative, and upper endoscopy showed an ulcerated mass in the second/third portion of the duodenum worrisome for duodenal adenocarcinoma (Figure 2). The mass was not bleeding at the time of the endoscopy. A biopsy of the mass done during endoscopy was inconclusive and showed cellular debris. The patient stabilized and stopped bleeding and was discharged home. She was electively seen at the hepatobiliary surgery clinic where additional staging workup was negative, including tumor markers. The patient's personal and family history were noncontributory. A pancreaticoduodenectomy was recommended based on the suspicion for a duodenal adenocarcinoma and was successfully performed. She had an uneventful hospital stay and was discharged home on postoperative day five. Her final pathology revealed a duodenal gangliocytic paraganglioma eroding into the pancreas, and all lymph nodes were negative for tumor. She was seen eight months postoperatively and was still free of disease.

## 3. Discussion

Duodenal GPs were described initially by Dahl et al. in 1957, and they were given their current name in 1971 by Kepes et al. [4, 14]. After the WHO introduced the new classification of gastrointestinal NETs, there has been an argumentative question as to classify GPs as low-grade NETs or allow them their independent entity based on better clinical behavior and better prognosis [9, 11, 12].

The clinical presentation of duodenal GPs varies, with upper gastrointestinal bleeding occurring in almost half of the patients (as in the case of our patient), while 15% present with anemia [15]. Abdominal pain, obstructive duodenal symptoms, pancreatitis, and abnormal incidental findings on axial abdominal imaging account for the rest of the clinical presentations [16, 17]. The local tumor symptoms are likely due to its predilection to the second portion of the duodenum in 90% of the cases [15]. It affects patients of all ages with an average age of diagnosis of 52 years, and there is a little more preponderance towards occurrence in males [15].

**Figure 3:**
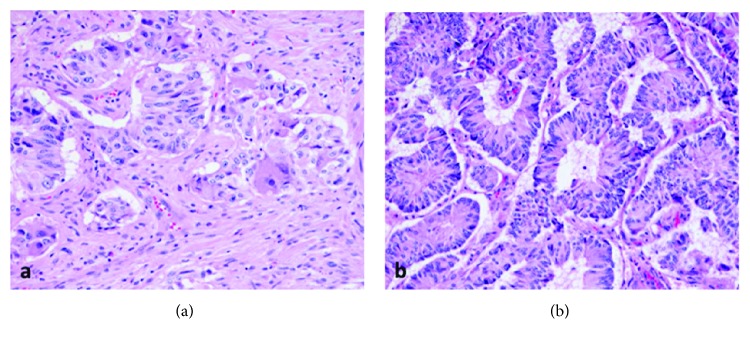
Hematoxylin and eosin (H&E) slides of our case report specimen, seen under low- and high-power magnification in (a) and (b), demonstrating the presence of three different cell types: epithelioid cells, spindle cells, and ganglion-like cells.

**Figure 4:**
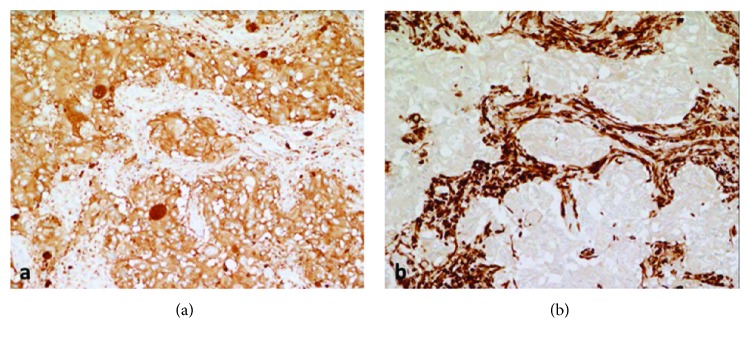
Pathology slides stained with immunohistochemistry. Synaptophysin stain, shown in (a), demonstrates epithelioid-type cells. Spindle cells stain positive for S100 in (b).

GP are composed of three cell types: (1) epithelioid cells resembling well-differentiated neuroendocrine tumors or paraganglioma in both cytologic and architectural features; (2) spindled cells resembling those seen in peripheral nerve sheath tumors; and (3) ganglion-like cells with characteristic abundant cytoplasm and prominent nucleoli (Figure 3). The cell types exhibit differential immunohistochemical staining patterns as well. Epithelioid cells are positive for neuroendocrine markers such as synaptophysin and chromogranin and are frequently positive for keratins (Figure 4(a)). Ganglion-like cells are positive only for synaptophysin. Spindle cells are positive for S100 (Figure 4(b)). The cell components can vary in distribution, delineation, and relative proportions within a tumor. There can be an intimate admixture as shown in Figure 3(a), as well as regions composed predominately of one cell type which can be essentially identical in morphology to a well-differentiated NET (Figure 3(b)). This heterogeneity can potentially lead to misdiagnosis of GP, particularly on biopsy, if the other cellular components of GP are not recognized.

Duodenal GPs were initially viewed as benign behaving tumors of the duodenum; however, there has been an increasing incidence of reports about lymph node metastasis and distant organ metastasis [7, 18, 19]. There has even been a report of a mortality directly related to a case of metastatic duodenal GP after resection [6]. Surgical resection and endoscopic resection were the only two curative methods used historically in treating these tumors, and the permanent pathological examination was the gold standard to obtain a correct and precise diagnosis. There was only one report of a case that received adjuvant radiation [20]. Local recurrence after resection has been extremely rare and was reported only once in the literature [21]. Appropriate treatment of duodenal GPs is still controversial. Some authors suggest that local endoscopic or surgical resection is sufficient due to the very low rate of lymph node metastasis and local recurrence. Other authors report the importance of a radical resection with a pancreaticoduodenectomy for a better lymph node basin clearance and prevention of the low number, yet, clinically significant metastatic rate [12, 15, 22].

## 4. Conclusion

Duodenal GP are rare tumors of the second portion of the duodenum that present with upper gastrointestinal bleeding and local obstructive symptoms of surrounding organs. They are classified as rare neuroendocrine neoplasms of the gastrointestinal tract and are usually considered benign and assigned an excellent prognosis. Appropriate methods of treatment are still controversial but range from endoscopic submucosal resection to pancreaticoduodenectomy.
